# Secondary prevention of preeclampsia

**DOI:** 10.3389/fcell.2025.1520218

**Published:** 2025-02-07

**Authors:** Muhammad Ilham Aldika Akbar, Roudhona Rosaudyn, Khanisyah Erza Gumilar, Renuka Shanmugalingam, Gustaaf Dekker

**Affiliations:** ^1^ Department Obstetrics and Gynecology, Faculty of Medicine, Universitas Airlangga, Surabaya, Indonesia; ^2^ Department Obstetrics and Gynecology, Universitas Airlangga Hospital, Surabaya, Indonesia; ^3^ Department Obstetrics and Gynecology, Dr. Soetomo General Academic Hospital, Surabaya, Indonesia; ^4^ Graduate Institute of Biomedical Science, China Medical University, Taichung, Taiwan; ^5^ Department of Renal Medicineand, Liverpool Hospital, Sydney, NSW, Australia; ^6^ Department Obstetrics and Gynecology, Lyell McEwin Hospital, University of Adelaide, Adelaide, SA, Australia

**Keywords:** preeclampsia, high risk pregnancy, prevention, maternal death, maternal health

## Abstract

Preventing preeclampsia (PE) is crucial for the wellbeing of the mother, fetus, and the neonate with three levels: primary, secondary, and tertiary. Secondary prevention involves pharmacological therapies aimed at stopping the disease’s progression before clinical signs. The predominant approach currently employed is the daily administration of low dose Aspirin and calcium. PE is a multifaceted illness characterized by syncytiotrophoblast (STB) stress, leading to endothelial dysfunction and systemic inflammation. Various subtypes of PE, in particular early-onset PE (EOP) and late-onset PE (LOP), have different pathophysiological pathways leading to STB stress and also different perinatal outcomes. Low-dose Aspirin (LDA) has been shown to be beneficial in lowering the occurrence of EOP, especially when started before 16 weeks of pregnancy. Calcium supplementation is advantageous for women with poor dietary calcium intake, reducing endothelium activation and hypertension. Low molecular weight heparins (LMWH), have pleiotropic effects, besides their anticoagulant effects, LMWH have significant anti-inflammatory effects, and have a potential restricted use in patients with history of prior severe placental vasculopathy with or without the maternal preeclamptic syndrome. Pravastatin and other statins have shown positive results in lowering preterm PE and improving outcomes for both the mother and baby. Proton pump inhibitors (PPIs) have shown potential in lowering soluble FMS-like tyrosine kinase-1 (sFlt-1) levels and enhancing endothelial function, but clinical trials have been inconsistent. Metformin, primarily used for improving insulin sensitivity, has potential advantages in decreasing PE incidence due to its anti-inflammatory and vascular properties, particularly in morbidly obese women. Nitric oxide (NO) donors and L-arginine have been shown to effectively reduce vascular resistance and improving blood flow to placenta, potentially reducing PE risk. In conclusion, various pharmacological treatments have the potential to prevent secondary PE, but their effectiveness depends on underlying risk factors and intervention time. Further research is needed to determine the optimal (combination) of method(s) for the individual patient with her individual risk profile.

## Introduction

Prevention of PE would represent a breakthrough in medicine. The general term prevention has 3 different connotations: primary, secondary, or tertiary. Primary prevention means avoiding occurrence of a disease. For PE this would be restricted to public health education efforts to reduce the rate of obesity and recommendations on having longer periods of sexual relationships prior to conceiving (Dekker and Robillard, 2021; Robillard et al., 2019). Secondary prevention in the context of PE implies breaking off the disease process before emergence of clinically recognizable disease–the focus of this review. Tertiary prevention means prevention of complications caused by the disease process, and is thus more or less synonymous with treatment (Dekker and Sibai, 2001).

The focus of this review is on secondary, primarily pharmacological, prevention of preeclampsia. Starting with a discussion on LDA going back to the mid-1980s, calcium supplementation starting in the 1990s, and followed by more recent preventative attempts like pravastatin, metformin, LMWH, PPI’s, and NO donors/L-arginine. Since secondary prevention typically targets one or more of the important pathogenetic/pathophysiologic pathways this review will start with a short summary of current understanding of this heterogeneous syndrome.

## Preeclampsia: a heterogeneous syndrome

Over many years, the late, Prof Chris Redman, one of the most influential PE researchers, has stressed the importance of not approaching PE as a single disease entity but as a heterogeneous syndrome (Redman, 2014). It is now abundantly clear that different pathways lead to the final common pathway of STB stress. The STB being a syncytium, cannot repair itself and ages (senescence) quite in contrast with, for example, liver parenchymal cells. “Premature aging,” an intrinsically inflammatory process, and the STB stress result, amongst other factors, in positive stress signals like increase sFlt-1and sEng and a negative stress signal (decrease PLGF) accompanied by degrees of systemic inflammation (Redman et al., 2022). The imbalance between PLGF and sFlt-1 appears to be one of the leading causes of the well-known endothelial cell disease with a drop in NO synthesis and the well known prostacyclin (PGI2) and Thromboxane A2 (TXA2) imbalance. STB stress (danger signal) will trigger oxidative stress, and the inflammatory cascade leading to an imbalance between pro-inflammatory and anti-inflammatory Th1 cells. Excessive production of pro-inflammatory cytokines, such as IL-6 and TNFα, further, affects the endothelium, not only by decreasing release of aforementioned vasodilators (PGI2 and NO) but also by the increased expression of endothelial cell adhesion molecules like immunoglobulin-like adhesion molecules, integrins, cadherins and selectins. Endothelial dysfunction and the systemic inflammation lead to vasoconstriction, platelet aggregation (TXA2), and increased vascular permeability. (Phipps et al., 2016; Maynard et al., 2005; Young et al., 2010; Matsubara et al., 2015; Anto et al., 2018; Dekker and Sibai, 1998).

The heterogeneity of the syndrome is based on the different pathways leading to STB stress. In the classic type of PE, so called early-onset PE (EOP) (PE leading to mostly iatrogenic preterm birth <34 weeks), STB stress is caused by superficial cytotrophoblast (CTB) invasion in the about 100 spiral arteries. This superficial CTB invasion leads to poorly modified spiral arteries and subsequently pulsatile high velocity damaging bloods flows in the intervillous space. Ongoing lack of spiral artery modification later leads to intermittent ischemia/reperfusion and oxidative stress adversely affecting the STB. The currently much more common phenotype of PE, is late-onset PE (LOP), i.e., PE leading to birth after 34 weeks (Staff and Redman, 2018a; Redman, 2017). Typically, in disease close to term there is no problem with original placentation, the STB stress more relates to chronic cardiometabolic conditions also associated with systemic inflammation (Chris Redman introduced the term “metaflammation”) (Roberts et al., 2017). EOP is typically associated with abnormal uterine artery Doppler flow patterns, fetal growth restriction (FGR), and adverse consequences for both the mother and the newborn. While LOP patients typically have normal uterine artery Doppler flow patterns and more favourable perinatal outcomes, patients still may experience major maternal morbidity if not recognised and appropriately managed the risk of maternal death (Valensise et al., 2008) (Staff and Redman, 2018b).

## Secondary prevention of preeclampsia

### Low-dose aspirin (LDA)

With the discovery that there was an imbalance between TXA2 and PGI2 in PE, it was reasonable to evaluate whether LDA would be effective for PE prevention. Aspirin, a non-selective COX inhibitor, at a low dose reduces TXA2 levels without reducing PGI2 levels due to the first pass effect (liver de-acetylates aspirin for 90%–95%) and the fact that platelets being without a nucleus cannot resynthesize COX (Ahn and Hwang, 2023). It is important to note that although Aspirin is a non-selective COX inhibitor, the dose of Aspirin may affect the effect on COX1 vs. COX2. Platelet inactivation occurs by inhibiting both COX-1 and COX-2, which in turn inhibits the production of TXA2. COX-1 is an enzyme that is present in all tissues at all times, whereas COX-2 is only produced in reaction to reactive oxygen species, cytokines, endotoxins, or growth factors during inflammatory circumstances (Faki and Er, 2021). The COX enzyme catalyzes the conversion of arachidonic acid into prostaglandin H2 (PGH2). PGH2 can then be further transformed into TXA2, PGI2 or PGE2, or other prostaglandins depending on the cell type and tissue (Mangana et al., 2021). TXA2 participates in platelet aggregation, vasoconstriction, and as a stimulant for smooth muscle cell growth. Conversely, PGI2 exhibits the contrary effect to TXA2 (Anto et al., 2018). When administered in low dosages, Aspirin specifically inhibits the activity of COX-1. However, when given in high doses (not applicable in the obstetric preventative context), aspirin inhibits the actions of both COX-1 and COX-2 (Shanmugalingam et al., 2019a).

The first double blind randomized clinical trial was published in 1986 by Wallenburg and Dekker (Dekker, 1989; Wallenburg et al., 1986). A large group of low-risk nulliparous pregnant women had an angiotensin II infusion test; 46 normotensive women at 28 weeks’ gestation were judged to be at risk for PE by increased blood pressure response to infused angiotensin II. Twelve of 23 women taking placebo developed PE, whereas only 2 of 21 women on 60 mg of Aspirin developed PE (83% decrease). Just prior to this double blind RCT, Beaufils et al. published a study in a group of just 102 patients with a historical risk of PE and/or FGR. In this unblinded study, patients were randomly allocated to receive 300 mg dipyridamole plus 150 mg of Aspirin; in the treatment group no cases of preeclampsia vs. 6 (8.5%) in the no-treatment group (Beaufils et al., 1985). It is not clear why 150 mg was chosen–but this study is still of historical interest, since the 150 mg was also used in the more recent ASPRE trial (Rolnik et al., 2017).

The following convoluted road that led to the current ongoing use of LDA in the prevention of PE is detailed in an elegant review by Scott Walsh and Jerome Strauss (Walsh and Strauss, 2021). A plethora of clinical trials followed, reporting varying degrees of effectiveness of LDA treatment. Two large multicenter intent-to-treat studies were conducted in nulliparous pregnant women given 60 mg/day of aspirin by the NICHD Maternal-Fetal Medicine Unit Network and the Collaborative Low-dose Aspirin Study in Pregnancy (CLASP) trials (Sibai et al., 1993; CLASP: a randomised trial of, 1994). Only modest decreases in the incidence of PE were found. The MFM Unit Network study reported no improvement in perinatal morbidity and a possible increased risk of placental abruption. Interest in LDA declined after the MFM Network Unit and CLASP studies due to the existing concerns about placental abruption and the small beneficial effects of LDA.

Real interest in LDA re-emerged by the re-analysis of all RCT by Roberge showing a massive reduction in the PE rate (OR 0.47) with LDA of at least 100 mg (virtually all these studies used 100 mg) started prior to 16 weeks’s gestation (Roberge et al., 2018). Finally, the ASPRE study by Rolnik et al., studying the effect of 150 mg of Aspirin (the old Beaufils dose) in patients with a high risk first trimester screen for PE as introduced by Kypros Nicolaides (Rolnik et al., 2017), demonstrated that LDA at a dosage of 150 mg per day from 11 to 14 weeks of pregnancy until 36 weeks can decrease the likelihood of developing PE. In the LDA group, only 1.6% of patients experienced preterm PE, compared to 4.3% in placebo group (62% reduction). There were no significant differences in terms of maternal complications during pregnancy or adverse impacts on the fetus as compared to the placebo group (Rolnik et al., 2017). Interestingly, in a *post hoc* analysis of the ASPRE trial, Poon et al. demonstrated that 150 mg of Aspirin does not prevent superimposed PE in patients with chronic hypertension, but may reduce “placental” preterm birth (Poon et al., 2017). A Cochrane review of 77 studies demonstrated that LDA decreased the likelihood of preterm birth decreased by 9% and fetal death by 15%. The efficacy of LDA is contingent upon the adherence of patients to the prescribed medication regimen, which has a success rate ranging from 76% to 90% in the prevention of PE (Rolnik et al., 2017; Brownfoot and Rolnik, 2024; Chang et al., 2023a). It is important to acknowledge that in wealthy nations where aneuploidy screening is standard, the FMF screening algorithm may prove cost-effective; however, this algorithm may not be universally applicable, especially in poor and resource-limited countries, due to its expensive costs and the requisite expertise and manpower needed to conduct high-quality uterine artery Doppler assessments around 12 weeks of gestation (Poon and Nicolaides, 2014). Moreover, in these contexts, the availability of serum indicators such as PAPP-A and PlGF may be significantly limited.

The dose of LDA is still a major controversial topic, it is important to note that in the Roberge et al. systematic review, the OR for developing PE was also 0.47 – virtually all the studies in this review used 100 mg of aspirin, i.e., similar efficacy as in the ASPRE trial (Roberge et al., 2018). Recent meta-analysis and pharmacokinetic studies, however, continue to contribute towards a growing body of evidence that favours the use of 150 mg daily (Ghesquiere et al., 2023; Shanmugalingam et al., 2019b)Nevertheless, current RCTs comparing the efficacy and side effect of various doses of Aspirin will provide better clarity on the optimal dose of aspirin in preventing PE (Brito et al., 2019; Sinha et al., 2023).

**FIGURE 1:**
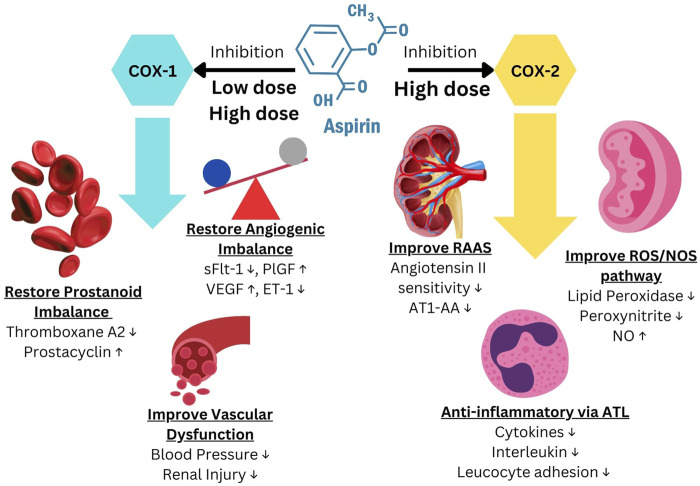
Aspirin mechanism of action to prevent Preeclampsia. Modified from Shanmugalingam et al. (2020), Mirabito Colafella et al. (2020).

Research over the past decade has shown that the effect of LDA involves much more than just correcting the PGI2/TXA2 imbalance. The fact that LDA only prevents EOP and has no effect on LOP clearly indicates the importance of LDA affecting placentation/TB function. The mechanism of COX-2 inhibition can improve the RAAS, ROS/NOS pathways, restore the angiogenesis balance, vascular function, and generate the substance 15-epi-Lipoxin A4, which possesses potent anti-inflammatory characteristics (Shanmugalingam et al., 2020). All of these effects may contribute via different pathways in the prevention of PE (Figure 1) (Shanmugalingam et al., 2020; Mirabito Colafella et al., 2020).

LDA is considered to be efficacious in preventing PE when administered at a dosage of ≥100 mg (preferably 150 mg), taken at night, and initiated before the 16th week of pregnancy and continued until the 36th week of pregnancy or until delivery (Brownfoot and Rolnik, 2024; Shen et al., 2021). ACOG, ISSHP, FIGO, SOMANZ, and NICE advise administering LDA to pregnant women who are at a high risk of developing PE, however, the recommended doses and time of initiation differs among these guidelines (Chang et al., 2023b; Bokuda and Ichihara, 2023) (Table 1).

**TABLE 1:** Comparison of recommendations on the use of aspirin in prevention of preeclampsia.

Organization	Indication	Timing	Dose
ACOG 2018	- Recommended for high risk pregnant women - Recommended for women with ≥1 moderate risk factors	Start from 12 to 28 weeks gestation (best at < 16 weeks), continued until delivery	81 mg/day
ISSHP 2018	- Recommended for high risk pregnant women	Start from <20 weeks gestation (best at < 16 weeks)	75–162 mg/day
NICE 2019	- Recommended for high risk pregnant women - Recommended for women with ≥1 moderate risk factors	Start from ≥12 weeks gestation, continued until delivery	75–150 mg/day
FIGO 2019	- Recommended for high risk pregnant women	Start from 11 to 14 weeks, and continued until 36 weeks, delivery, or onset of preeclampsia	150 mg/day
SOMANZ 2023	- Recommended for high risk pregnant women	Start prior of 16 weeks, and stop between 34 weeks until delivery, based on individualised judgement	150 mg/day
POGI 2016	- Recommended for high risk pregnant women	Start from <20 weeks gestation	75 mg/day

### Calcium and vitamin D

**FIGURE 2:**
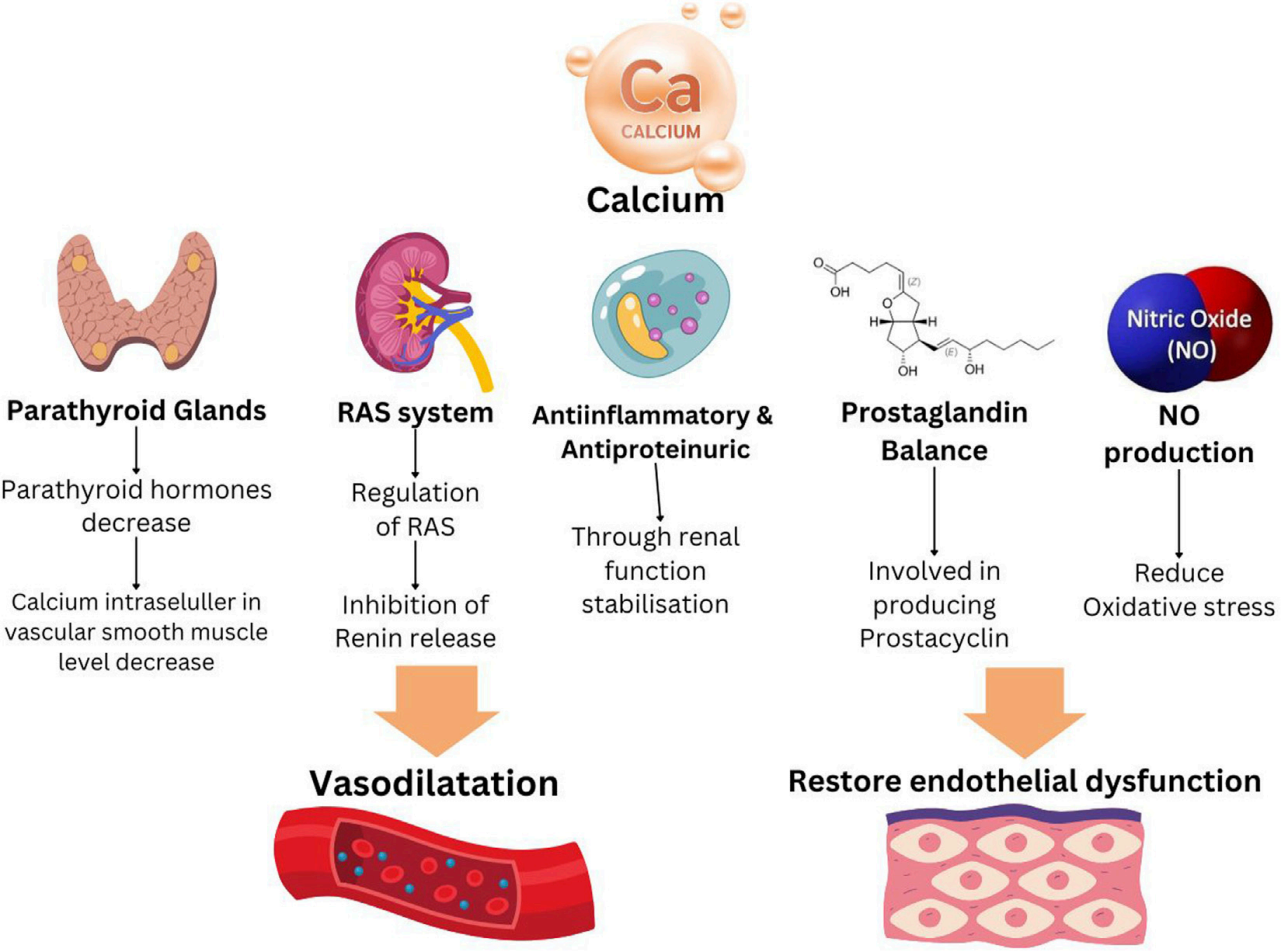
Mechanism of action of calcium in preeclampsia prevention.

Oral calcium supplementation is recommended as an additional preventative intervention in women with inadequate dietary calcium intake (<1 g/day) (Magee et al., 2022; Hofmeyr et al., 2019) Calcium minimises endothelial cell activation through anti-inflammatory cytokines and upregulation of NO (Cabral-Pacheco et al., 2020; López-Jaramillo et al., 1995). Hypocalcemia can lead to activation of parathyroid glands, which in turn promotes the secretion of renin. Elevated intracellular calcium levels will induce vasoconstriction (Figure 2) (van Gelder et al., 2022). A Cochrane study demonstrated evidence from 27 randomized controlled trials supporting the efficacy of calcium supplementation in preventing PE and preterm birth. Additionally, calcium supplement reduce the risk of maternal mortality and significant complications associated with high blood pressure during pregnancy (Hofmeyr et al., 2018). This is specifically intended for women who are following low calcium diets (Hofmeyr et al., 2018). This discovery is supported by the World Health Organization (WHO), which demonstrated that administering calcium to pregnant women in regions with calcium deficiency can effectively lower the risk of hypertension during pregnancy. According to a review by Brownfoot et a administering calcium has limited impact on reducing the likelihood of PE but it plays a crucial role in mitigating severe consequences associated with PE, such as eclampsia, severe gestational hypertension, and neonatal mortality (Brownfoot and Rolnik, 2024).

Nonetheless, Brownfoot’s perspective has faced numerous challenges from different studies. Two systematic reviews report that calcium supplementation, as compared to a placebo, resulted in a 51%–55% decrease in the development of preeclampsia (Hofmeyr et al., 2018; Woo Kinshella et al., 2022). The advantages of supplementing remain consistent regardless of the dosage, risk of preeclampsia, time of calcium delivery, or co-interventions, particularly vitamin D. Nevertheless, the efficacy of calcium is restricted in people with inadequate initial calcium consumption. Administering calcium was linked to a slight 0.2% increase in the probability of developing HELLP syndrome, but it also resulted in a 1.0% decrease in the occurrence of death or severe maternal morbidity. While calcium does not definitively decrease the occurrence of preterm PE, it does lower the prevalence of preterm birth and infants with low birth weight (Woo Kinshella et al., 2022). A recent meta-analysis, which included 26 randomized controlled trials with a total of 20,038 participants, revealed that the administration of calcium resulted in a 49% reduction in the risk of PE and a 30% reduction in the risk of gestational hypertension when compared to a placebo. In addition, there was a propensity to decrease the occurrence of preterm labour, labour induction, small for gestational age, low birth weight infants, perinatal mortality, and maternal mortality in the group that received calcium supplementation (Jaiswal et al., 2024).

Administering low doses of calcium, either alone or in combination with other nutrients, has been shown in multiple studies to decrease the occurrence of preeclampsia. Research indicates that the administration of high doses of calcium can effectively lower the likelihood of elevated blood pressure. Overall, calcium administration generally lowers the incidence of PE. However, this impact is most significant in pregnant women who have a poor intake of calcium. Pregnant women who have a low intake of calcium (<800 mg/day) are urged to consume either calcium replacement (<1 g elemental calcium/day) or calcium supplementation (1.5–2 g elemental calcium/day) in order to decrease the likelihood of developing preeclampsia (Poon et al., 2019).

New evidence indicates that the dosage of calcium does not impact its efficacy in preventing PE. Kinshella et al. performed a network meta-analysis (NMA) to assess the efficacy of low dose (<1 g/day) and high dose (>1 g/day) calcium supplementation in the prevention of PE. The evaluation of calcium dose by the NMA included 25 trials with a total of 15,038 participants. In contrast, the meta-analysis included 30 trials with a total of 20,445 women. Calcium supplementation at both high and low doses effectively reduced PE, with a relative risk (RR) of 0.49 and 95% confidence intervals (CI) of 0.36–0.66 and 0.49%, 95% CI 0.36–0.65, respectively. According to the NMA, there was no clear difference in the impact of high-dose calcium compared to low-dose calcium (RR 0.79%, 95% CI 0.43–1.40). The Cochrane research also endorses the use of either high or low doses of calcium to prevent PE. Nevertheless, the administration of a low dosage of calcium did not exhibit a distinct impact on preterm birth, stillbirth, or mortality prior to departure from the hospital (Hofmeyr et al., 2018). Calcium was found to be similarly effective regardless of the risk of PE in early pregnancy, the simultaneous use of vitamin D, or the date of calcium initiation (Woo Kinshella et al., 2022).

In conclusion, the WHO (2018) recommends for daily oral calcium supplementation at a dosage of 1.5–2 g (elemental calcium) in populations with insufficient dietary calcium consumption to mitigate the risk of PE, irrespective of individual preeclampsia risk factors (WHO, 2018). The most recent recommendations (2020) propose for calcium supplementation prior to pregnancy (preconception) to reduce the risk of PE, but within the context of scientific research (WHO) (WHO, 2024). A 2019 multicountry trial (n = 1355) compared 500 mg calcium or placebo daily from enrolment before pregnancy to 20 weeks of gestation, then 1.5 g calcium/day in both groups. The intervention did not reduce PE overall, but participants with compliance of more than 80% from the last prepregnancy visit to 20 weeks had a statistically significant effect (RR = 0.66, 95% CI: 0.44–0.98; P = 0.037). This is the basis of WHO’s latest recommendations (Hofmeyr et al., 2019). The summarized recommendation for calcium supplementation during pregnancy to prevent preeclampsia is presented in Table 2.

**TABLE 2:** Calcium suplementation during pregnancy to prevent preeclampsia.

Mechanisms of action	- Vasodilators caused by - Inhibition release of renin from parathyroid glands - Reduce intracelullar calcium level in vascular smooth muscle - Upregulations of NO - Anti-inflammatory properties - Inhibiton of endothelial activation (López-Jaramillo et al., 1995; van Gelder et al., 2022; Omotayo et al., 2016)
Target population	Pregnant women with low dietary calcium consumption (<800 mg/day) (WHO, 2018; Omotayo et al., 2016)
Calcium type	Calcium carbonate (Omotayo et al., 2016)
Doses	- WHO recommends 1.5–2 g daily (WHO, 2018) - Some evidence suggest dose less than 1 g/day (low dose) may be beneficial (Omotayo et al., 2016)
Timing	Start as early as possible in pregnancy, limited evidence suggest to start before pregnancy (pre-conception) (Hofmeyr et al., 2019; Poon et al., 2019)

PE has been linked to hypovitaminosis D (Bodnar et al., 2007; Ilham et al., 2019). Multiple hypotheses propose a relationship between vitamin D levels and the development of PE. Among these are vitamin D’s functions in the modulation of pro-inflammatory responses and the reduction of oxidative stress in PE, the promotion of angiogenesis through VEGF and gene modulation, and the reduction of blood pressure through the renin-angiotensin system (RAS) (Purswani et al., 2017). Nevertheless, the findings of numerous studies demonstrate contradictory outcomes for the efficacy of vitamin D in avoiding preeclampsia. The updated systematic review in Cochrane (2024) indicated that among eight studies, vitamin D supplementation compared to placebo for the prevention of PE demonstrated uncertain evidence (Palacios et al., 2019). In other systematic review by Purswani JM et al., showed that the evidence of the role of vitamin D in preventing PE is inconsistent (Purswani et al., 2017). These conclussion was taken mostly from observational study and only two RCT involved in this review. In a 50-year-old controlled experiment with 5,644 women, Olsen and Secher demonstrated a 31.5% reduction in the incidence of preeclampsia following multivitamin and mineral supplementation. This study wasn’t focused just on vitamin D as a preventive measure for PE, but rather included multivitamin and mineral supplements (Olsen and Secher, 1990). However in Alimoradi’s metanalysis including 19 studies, it was shown that the supplementation of vitamin D reduce the risk of PE for 39% (RR: 0.61; 95% CI: 0.47–0.78; *p* = 0.27) (Alimoradi et al., 2024). This was supported by AlSubai’s metanalysis including 10 RCT and 24 observational studies with the same result (OR: 0.50; 95% CI: 0.4–0.63; *p* = 0.00001) (AlSubai et al., 2023). In summary, the evidence regarding the utilization of vitamin D as a preventive measure for PE remains inconclusive. Nonetheless, there exists a certain potential in utilizing these agents for the prevention of PE, particularly within populations deficient in vitamin D.

### Low molecular weight heparin (LMWH)

**FIGURE 3:**
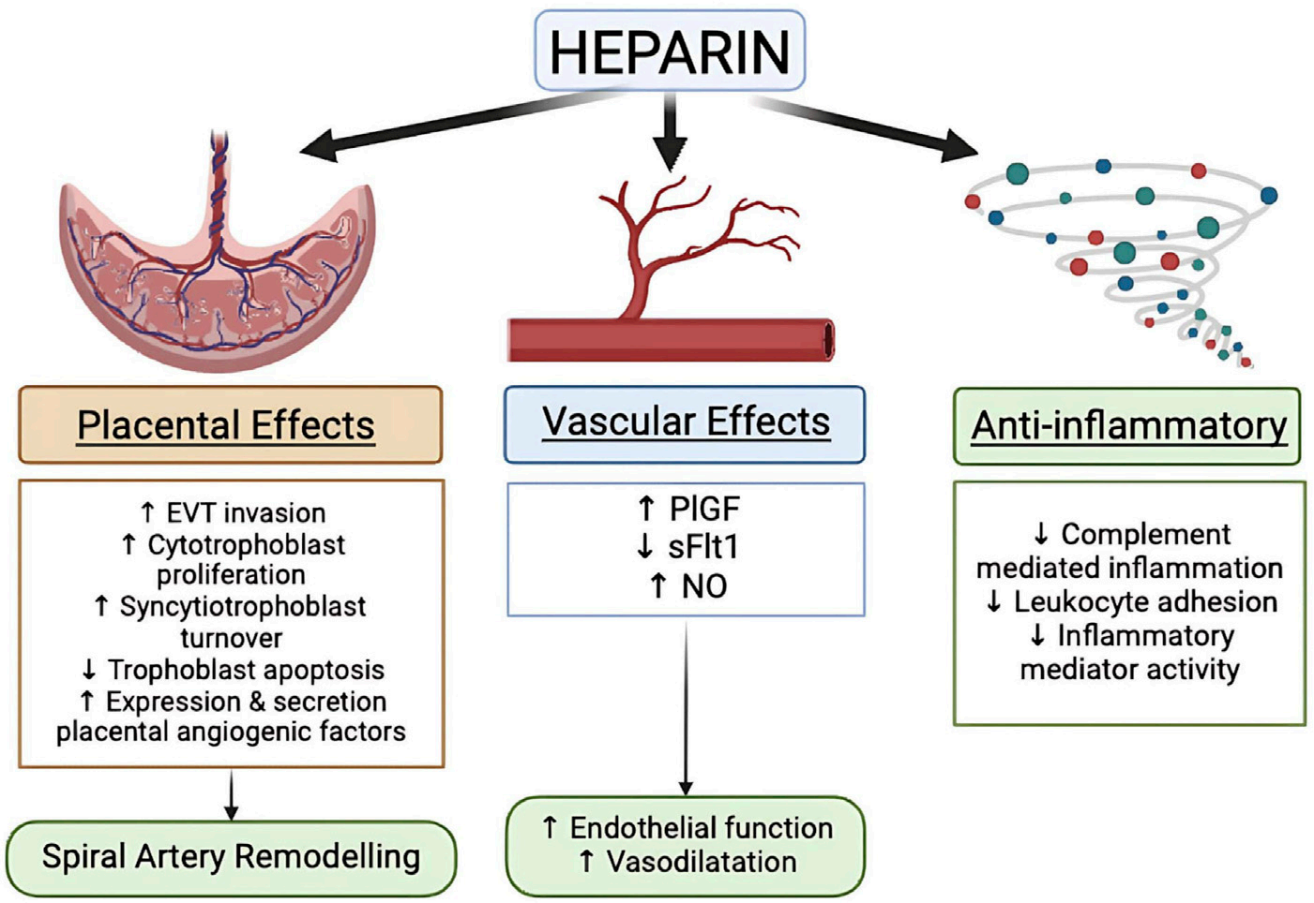
Heparin mechanism on the prevention of PE [modified from Wat et al. (2018a)].

Already in 1976, Bonnar and Redman contemplated whether or not there could be a place for heparin in the prevention of PE. Now 50 years later and after many trials, the role of LMWH in the prevention of PE still is still a topic of debate (Bonnar et al., 1976). Although heparin and the various LMWH’s are primarily known as anticoagulant agents, as a group they clearly also possess many anticoagulant-independent properties that may be relevant in the prevention of PE, including effects on placental, vascular and inflammatory function (Figure 3) (Wat et al., 2018a). A meta-analysis by Roberge et al. on 8 studies found that combined LMWH and LDA therapy is superior to LDA alone in preventing recurrent PE (relative risk [RR] 0.54, 95% CI 0.31–0.92) and SGA births (RR 0.54, 95% CI 0.32–0.91) (Roberge et al., 2016). A separate meta-analysis conducted by Rodger et al. also showed that LMWH–the most commonly prescribed heparin derivative, including dalteparin, enoxaparin, and nadroparin are effective in augmenting the preventive efficacy of aspirin as compared with LDA alone (14% *versus* 27%) (Rodger et al., 2016). However, LMWH alone does not appear to significantly reduce the rates of PE or SGA births, suggesting that is has synergistic effects with aspirin (Singh et al., 2024). Despite these promising findings, several recent large multicentre trials, such as the EPPI, HEPEPE and TIPPS trials, did not find similar beneficial effects of LMWH therapy for PE prevention.

The New Zealand (NZ) non blinded trial (EPPI) by Groom et al. examined the use of 40 mg of enoxaparin in 155 high-risk women; concerns about this trial would be that it included patients with BMI’s > 40. Also, the subgroup of patients with prior preterm PE (<36 weeks) was only 30 *versus* 38. Regarding the dose, we clearly miss good data on pharmacokinetics of LMWH in very obese pregnant women, also as addressed earlier, we still don’t know exactly what the main beneficial effect is of LMWH (Groom et al., 2016). The French study by Haddad et al. (HEPEPE) looked at placenta-mediated pregnancy complications. In an open label multicentre trial 124 patients received 100 mg of enoxaparin plus 100 mg of aspirin *versus* only aspirin. The rate of placenta-mediated complications was only modestly but not significantly reduced in the LMWH group 34.4% compared with 41% (relative risk 0.84, 95% CI 0.61–1.16, P = 0.29). This is an important study using a more adequate dose of enoxaparin. However, it should be noted that only 4 *versus* 7 patients had a history of early-onset PE, close to 50% in both groups were included for prior fetal losses <22 weeks (Haddad et al., 2016).

Furthermore, the use of LMWH for the prevention of PE carries more potential risk than the use LDA, such as bleeding and heparin-induced thrombocytopenia, although such risks were demonstrated to be minimal in recent randomized clinical trials (Arepally, 2017; Zullino et al., 2021). One of the primary limitations of these large trials is the inclusion of all patients with the preeclamptic syndrome with out consideration of the underlying etiology, thereby diluting the potential efficacy of LMWH therapy, which may benefit only a subset of patients. Therefore, further investigation is justified to evaluate the therapeutic potential of LMWH for the prevention of PE (McLaughlin et al., 2018).

A classic example is the very large well conduced multicentre TIPPS trial by Rodgers et al.; a trial that took 12 years to complete and eventually included 143 “high risk” patients receiving dalteparin and 141 placebo on top of LDA (Rodger, 2014). This ambitious study, where we can only admire the stamina of the researchers, tried to look at “everything,” prevention of venous thrombo-embolism, pregnancy loss and placenta-mediated complications. The TIPPS study did not show any benefit. But the authors failed to emphasize that close to 90% of patients were included for just having a simple thrombophilia like factor V, prothrombin gene or protein S deficiency. Clearly just having one of these thrombophilia does not require prophylactic treatment with LMWH (and importantly this was confirmed by the TIPPS study). Only 20 *versus* 25 had a history of PE (gestational age not even provided), as such the TIPPS study was very much underpowered to address the prevention of preterm PE (Rodger, 2014).

The most recent systematic review by Lemini et al. included 15 studies (also the aforementioned study by Haddad et al. and even the TIPPS study), with a total of 2795 participants. In high-risk women, treatment with LMWH in addition to LDA was associated with a reduction in the rate of PE, (OR 0.62; 95%: 0.43–0.90; P¼.010); SGA (OR 0.61; 95% CI 0.44–0.85) and perinatal death (OR 0.49; 95% CI 0.25–0.94). The authors of this review do emphasize their concerns about methodological quality of the studies ranged from moderate to very low owing to concerns about the risk of bias (double blinding not possible), type of patients included (e.g., TIPPS) and the variable dose of LMWH (Cruz-Lemini et al., 2022).

In summary, just having a thrombophilia does not warrant the use of prophylactic LMWH. The benefits of LMWH (similar to LDA) are clearly pleiotropic, and much more than just antithrombotic (Wat et al., 2018b). LMWH should not be used as a routine in the prevention of PE, but their use in combination with LDA has a defined place in preventing recurrent placental mediated complications (with or without PE) particular in the group of patients with documented prior placental vasculopathy.

### Pravastatin

**FIGURE 4:**
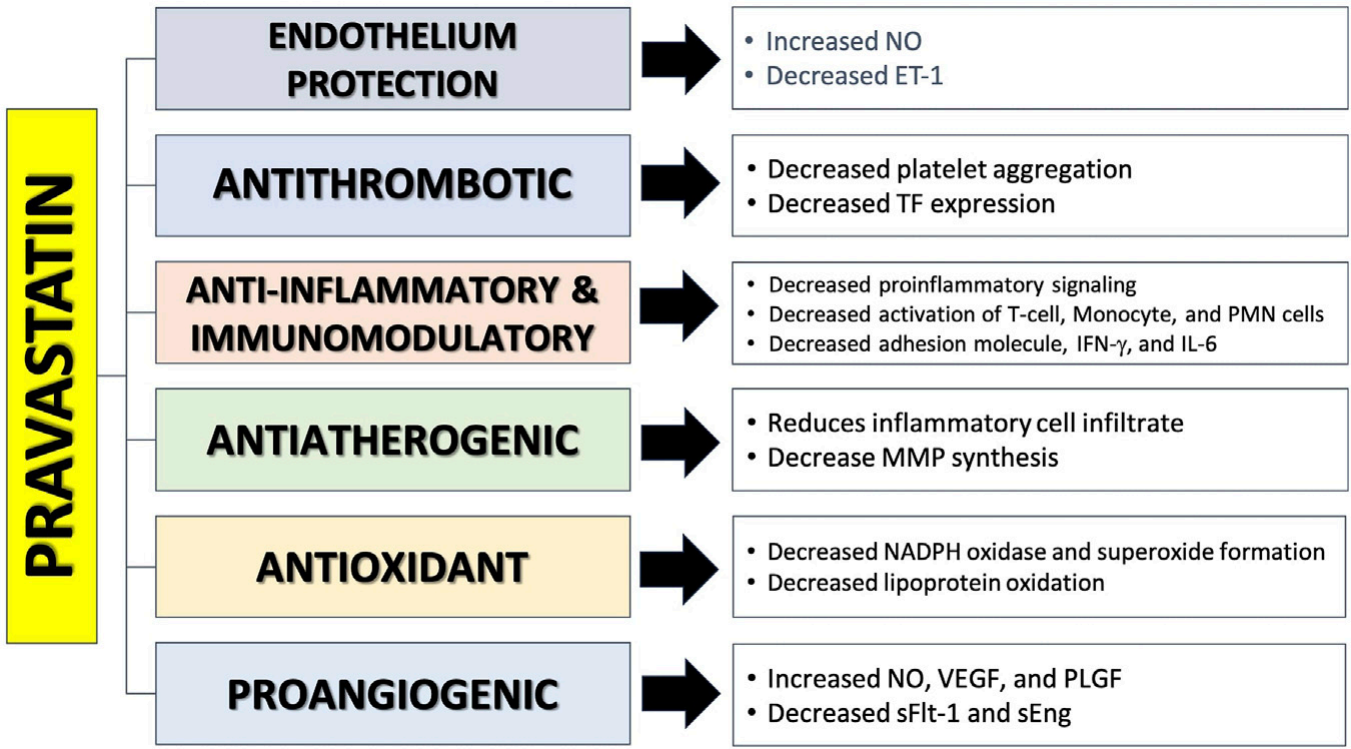
The mechanism of action of pravastatin in preventing preeclampsia (Katsi et al., 2017; Ramma and Ahmed, 2014; Vahedian-Azimi et al., 2021; Girardi, 2017; Tong et al., 2022a; Mészáros et al., 2023a; Akbar et al., 2021a; Akbar et al., 2024).

Statins are commonly utilized to reduce cholesterol levels and manage cardiovascular risks. Statins function as inhibitors of the enzyme HMG-CoA reductase, which is responsible for the production of 3-hydroxy-methylglutaryl coenzyme A (HMG-CoA)(ref). Statins are gaining prominence in studies as a potential preventive agent for PE. Laboratory studies, involving molecular analysis, animals, and preclinical research, have shown that statins have beneficial effects on many pathways involved in the development of PE (Katsi et al., 2017; Ramma and Ahmed, 2014; Ilham Aldika Akbar, 2021). The pleiotropic potentially beneficial effects of pravastatin in preventing PE are presented in Figure 4.

Pravastatin has been found to reverse angiogenic imbalance and placental hypoxia, characterized by elevated sFlt-1 expression, in experimental mice with preeclampsia (Ahmed and Ramma, 2015). The cause of this effect is believed to be the activation of the heme oxygenase 1/carbon monoxide (HO-1/CO) pathway. The treatment of statins in both *in vivo* and *in vitro* experiments resulted in an increase in the expression and transcription of HO-1 in endothelial cells, vascular smooth muscle, and other cells. HO-1 is a crucial antioxidant protein that plays a significant role in the process of converting heme into biliverdin, resulting in the release of carbon monoxide (CO) and ferrous ions (Fe^2+^) (Saad et al., 2014). Activation of this pathway suppresses the secretion of sFlt-1 and sEng from endothelial and placental cells, and is believed to promote the synthesis of VEGF and P1GF. In the end, the production of ET-1 will decrease and the levels of NO will increase as a result of decreased oxidative stress in endothelial cells (Akbar et al., 2021a; Saad et al., 2014; Akbar et al., 2022). Brownfoot et al. reported that pravastatin has the potential to decrease the release of sFlt-1 from isolated cytotrophoblast cells and human umbilical vein endothelial cells (HUVEC) acquired from preeclamptic patients (Brownfoot et al., 2015; Brownfoot et al., 2016a). Pravastatin is also able to reduce the expression of VCAM-1 and ET-1 and reduce leukocyte adhesion to endothelial cells. During a trial investigation with HUVEC, pravastatin had the least harmful effect when compared to simvastatin and rosuvastatin. All three statins shown efficacy in lowering ET-1 and sFLt-1, which are crucial variables contributing to endothelial dysfunction, during this experiment. High doses of simvastatin and rosuvastatin exhibit harmful effects on endothelial cells (Rodger, 2014; Cruz-Lemini et al., 2022).

**FIGURE 5:**
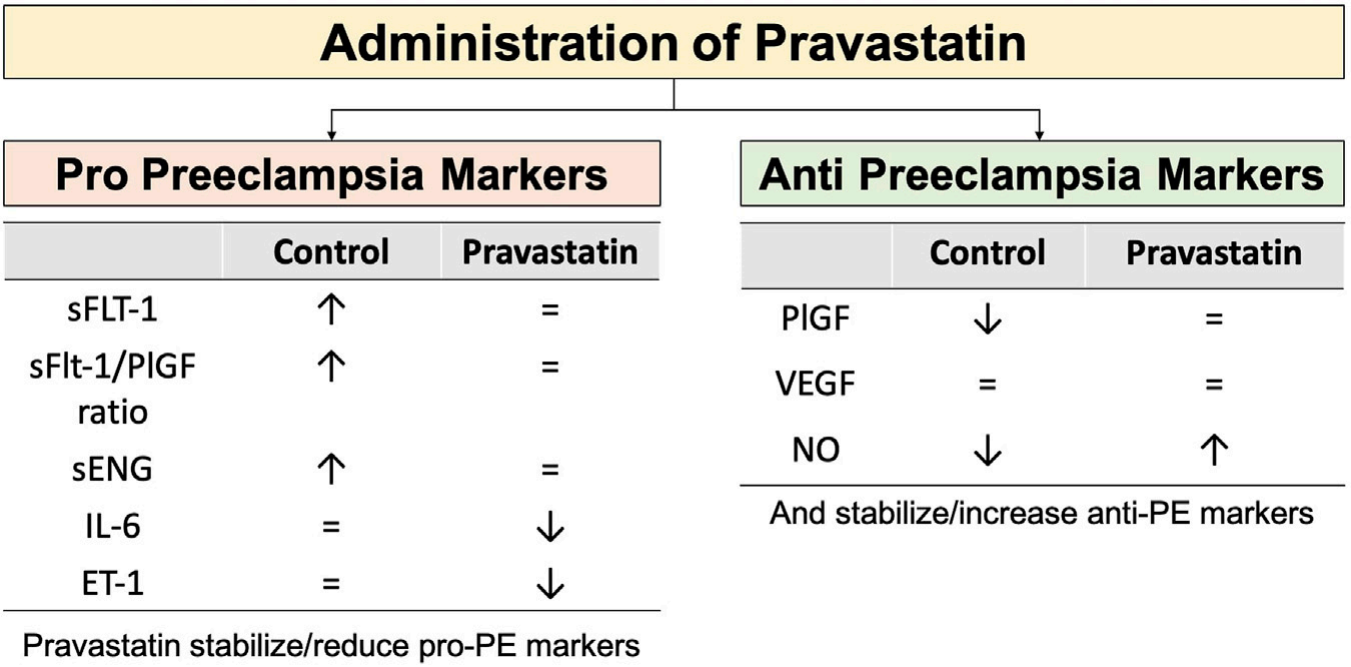
The effect of administration of Pravastatin in serum biomarkers changes in high risk PE pregnant women *versus* control. Pravastatin has the ability to stabilize or decrease the levels of pro-PE markers. In the control group, there is an observed increase in the levels of pro-PE markers. Pravastatin has the effect of stabilizing or increasing the levels of anti-PE markers. In the control group, there is a considerable fall in the levels of anti-PE markers. Pravastatin protects against alterations in biomarkers that contribute to the development of PE (Akbar et al., 2021a; Akbar et al., 2021b). Notes: ↑: increase level, ↓: decrease level, = : no significant changes.

Additional preclinical studies also suggested that pravastatin may have a preventive effect on PE due to its positive impact on maternal and placental blood vessels (Tong et al., 2022b). Costantine et al. conducted a small pilot randomized controlled trial (RCT) focussed on pharmacokinetics and side effects with a sample size of 20, and found a lower (non-significant) rate of PE in the pravastatin group (0 *versus* 4). Importantly, cord blood profiles were not different and pravastatin levels in cord blood were below detection level. Administration of pravastatin also improved the patient’s angiogenic profile by reducing levels of sFlt-1 and sEng, and boosting levels of PIGF (Costantine et al., 2016). The first sizeable (unblinded) multicenter RCT was conducted by Akbar et al. In this multicentre trial high risk patients with an estimated risk of 40% for developing PE were receiving LDA vs. LDA plus 20 mg bd pravastatin. In the Surabaya arm of this Indonesian INOVASIA study various PE biomarkers and cytokines were also examined. The biomarkers can be divided based on their action in the pathogenesis of PE: the anti-angiogenic factors (driving the development of PE), i.e., sFlt-1, sEng, sFlt-1/PlGF ratio, IL-6, and ET-1; and the pro-angiogenic factors (reducing PE risk), i.e., PlGF, VEGF, and NO (Akbar et al., 2021a). Pravastatin demonstrated the ability to stabilize fluctuations in levels of sFlt-1, PlGF, sFlt-1/PlGF ratio, and sEng when compared to the control group (Akbar et al., 2021b). The control group had a noteworthy rise in sFlt-1, sFLt-1/PlGF ratio, sEng, and PlGF, indicating alterations in the development of PE. In addition, Akbar et al. study shown that the administration of Pravastatin not only enhanced NO levels but also decreased IL-6 and ET-1 levels (Akbar et al., 2021a). Figure 5 provides an overview of all effects of pravastatin on the various biomarkers changes on Pravastatin administration based on INOVASIA study.

The overall results of the Indonesian multicentre RCT included 87 women in the treatment group and 86 women in the control group. The use of pravastatin greatly decreases the occurrence of preterm PE (odds ratio = 0.034; 95% CI0.2–0.91) and (mostly iatrogenic) premature birth (OR 0.340; 95% CI0.165–0.7). There was no effect on the overall PE rate, but because of the beneficial effects of pravastatin on preterm PE, administration of pravastatin also improved perinatal outcomes, such as increased Apgar scores and reduced incidence of low birth weight infants. Furthermore, there were no cases of congenital anomalies observed in the infants of mothers who were administered pravastatin (Akbar et al., 2022).

A recent systematic analysis conducted by Akbar et al. has shown that Pravastatin is linked to a decreased likelihood of PE (OR: 0.51; 95% CI: 0.29–0.90), preterm PE (OR: 0.034; 95% CI: 0.202–0.905), and preterm birth (OR: 0.31; 95% CI: 0.16–0.58). Pravastatin had no effect on the likelihood of developing PE with severe characteristics and having a small size for gestational age. Pregnant women who were administered pravastatin experienced improved perinatal outcomes, including mean higher birthweight, better Apgar scores, reduced NICU admission rates, shorter length of stay, and lower incidence of respiratory distress syndrome (Akbar et al., 2024). The findings of these studies suggest that Pravastatin has potential as a preventive treatment for PE, particularly preterm PE, as well as (iatrogenic) preterm labour. Additional long term follow up research is required to repeat these findings in different populations with particular focus on neurodevelopment milestones in pravastatin exposed infants.

### Metformin

**FIGURE 6:**
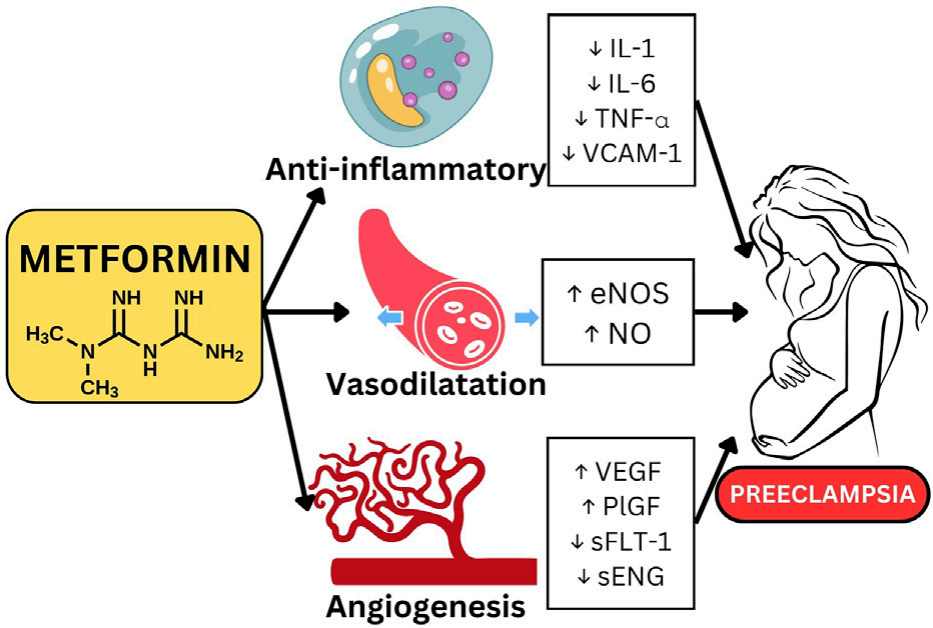
Metformin mechanism of action in reducing Preeclampsia risk.

Metformin is a drug to increase insulin sensitivity and reduce blood glucose levels. Metformin is commonly used as a treatment for Polycystic Ovary Syndrome (PCOS) and gestational diabetes mellitus during pregnancy. Experimental studies have demonstrated that Metformin exerts an influence on many parameters that contribute to the reduction of PE risk in animal models. Metformin is believed to inhibit nuclear factor kappa B (NF-kB) by activating the AMPK pathway, which leads to a decrease in the production of pro-inflammatory substances such IL-1B, IL-6, TNFα, IL-8, and IL-2. Additionally, metformin increases the activity of eNOS, which promotes the release of nitric oxide (NO) and prostaglandin E2 (PGE2). Another study shown that metformin effectively suppressed the activity of VCAM1 and ICAM1 within endothelial cells, leading to enhanced vascularization. Additionally, metformin increased the levels of matrix metalloproteinase 2 (MMP-2) and vascular endothelial growth factor (VEGF) (Brownfoot et al., 2016b; Poniedziałek-Czajkowska et al., 2021). In human studies, metformin has been found to decrease protein and gene expression from inflammatory endothelium cells, as well as VCAM-1, in individuals with diabetes mellitus and impaired glucose tolerance. In another research study, metformin was found to decrease the levels of sFlt-1 and sEng in human tissue, most likely by inhibiting the mitochondrial transport chain. This chain was found to be more active in placentas affected by preterm PE. Metformin has the ability to decrease endothelial dysfunction and enhance angiogenesis (Brownfoot et al., 2016b). The mechanism of action by which metformin prevents PE is displayed in Figure 6.

The impact of metformin on the probability of developing PE varies across studies, maybe due to variations in dosage (ranging from 500 to 3,000 mg/day), the presence of different underlying conditions (such as obesity, PCOS, gestational diabetic mellitus, or type 2 DM), or differences in the timing of medication starting (ranging from 6 to 36 weeks). Metformin has a notable impact on reducing the occurrence of PE in pregnant women who have morbid obesity. However, in pregnancies complicated by gestational diabetes mellitus, multiple studies have found no statistically significant difference in the occurrence of PE between the group receiving metformin and the control group. The metformin group did not experience a reduction in the risk of PE or gestational hypertension, indicating the ineffectiveness of metformin in this regard (Poniedziałek-Czajkowska et al., 2021). The EMPOWAR research was a clinical experiment that assessed the impact of providing metformin to pregnant obese women. The trial was randomized, double-blind, and placebo-controlled. The experimental cohort received a daily dosage of metformin ranging from 500 to 2,500 mg, starting at 12 weeks of age and continuing until delivery. No significant difference in the occurrence of PE was seen between the groups receiving metformin and placebo (Chiswick et al., 2015).

The meta-analysis conducted by Alqudah A et al. included 5 randomized controlled trials (RCTs) that compared metformin with placebo. The study indicated no significant difference in the risk of PE between the two groups RR = 0.86 (95% CI 0.33–2.26), p-value of 0.76. However, positive outcomes were observed in terms of lower maternal weight gain and a reduced risk of PE when compared to the insulin group (McDougall et al., 2022). Kalafat et al. conducted a meta-analysis of 15 randomized controlled trials (RCTs) and discovered that in women with gestational diabetes, the use of metformin was linked to a lower risk of pregnancy-induced hypertension compared to insulin. Additionally, there was a slightly lower risk of PE, but this reduction was not statistically significant. In obese women, the usage of metformin was found to have a minimal effect on reducing the incidence of PE, when compared to a placebo (Kalafat et al., 2018). In metaanalysis involving 35 studies, Metformin was associated with lower gestational weight gain (1.57 kg ± 0.60 kg; I_2_ = 86%, *p* < 0.0001) and likelihood of PE (OR 0.69, 95% CI 0.50–0.95; I_2_ = 55%, *p* = 0.02) compared to placebo (Tarry-Adkins et al., 2021). In another study, Metformin was reported to reduce the risk of abortion, preterm PE, preterm labor, and gestational HT (He et al., 2023).

Multiple studies have identified an increased risk of harm to the unborn child when metformin is used during pregnancy. Studies also indicate a correlation between the consumption of metformin during pregnancy and the occurrence of a small for gestational age fetus. The reason for this is believed to be that metformin influences the availability of nutrients and the growth of the fetus by inhibiting mitochondrial complex I. The fetus may experience cardiometabolic issues as a result of an imbalance between folic acid and vitamin B12. Therefore, it is advisable to take these B vitamins in conjunction with metformin administration (Verma and Mehendale, 2022). It should be note that the FDA categorizes Metformin as safe (category B)for pregnant women (Mészáros et al., 2023a). (Akbar et al., 2021b).

### Proton pump inhibitor

**FIGURE 7:**
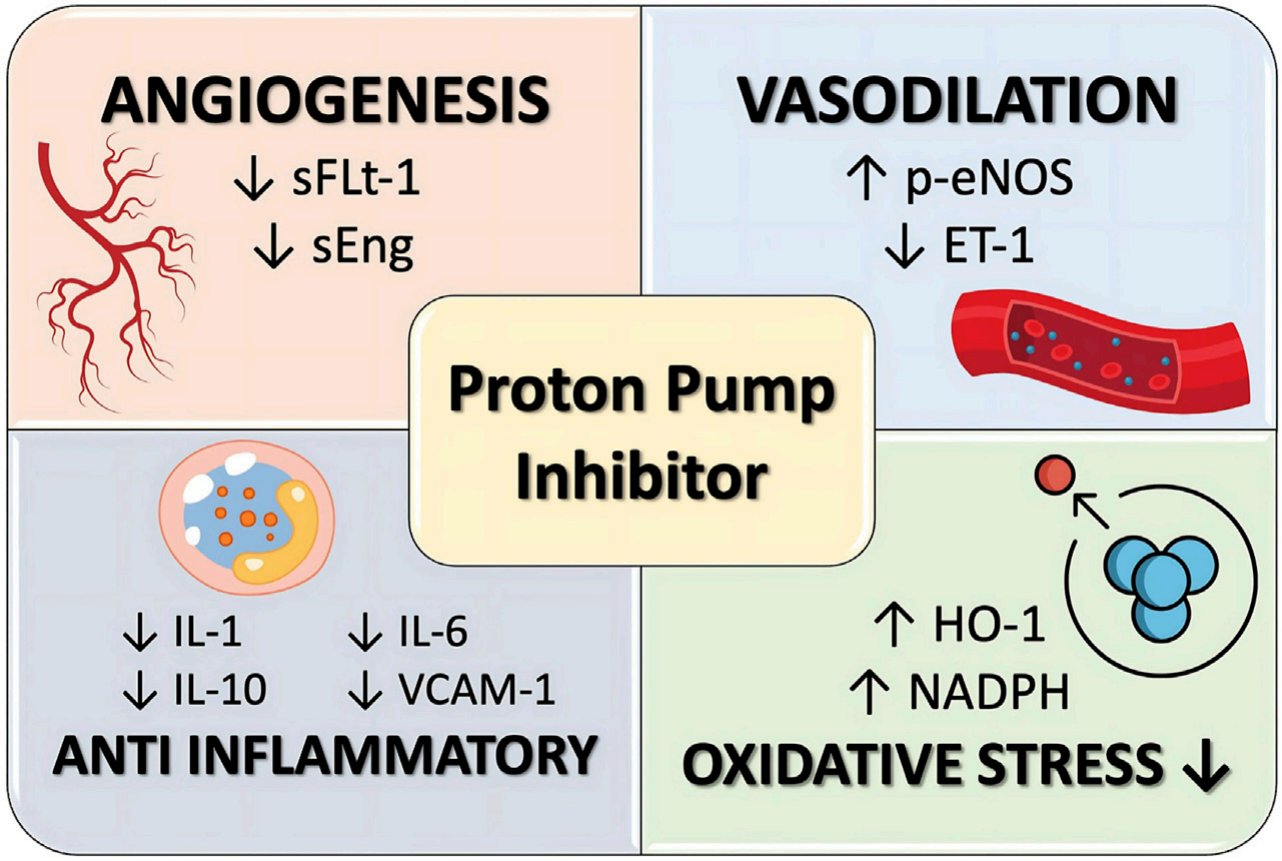
PPI mechanism of action in preeclampsia prevention.

Proton Pump Inhibitors (PPIs) hinder the activity of the hydrogen-potassium-ATPase pump located in the parietal cells lining the stomach, resulting in a decrease in the production of gastric acid. PPIs are often prescribed medications for the treatment of gastric reflux disease. PPI have been deemed safe for use by pregnant women according to a meta-analysis study (Matok et al., 2012). The impact of PPI on the prevention or treatment of PE is currently under investigation (Hastie et al., 2019). Experimental investigations have shown that PPIs have the ability to decrease sFlt-1 levels in animals (Gu et al., 2022). Onda et al. reported that the administration of PPI can decrease the production of sFlt-1 and sEng in several types of cells, including primary trophoblast cells, normal and preeclamptic placental cells, HUVECs, and primary uterine microvascular cells. Esomeprazole, the most powerful PPI, exerts a vasodilatory impact on blood vessels and reduces blood pressure by affecting endothelial cells (Onda et al., 2017). The study conducted by Saleh et al. shown a correlation between the use of PPI and a reduction in blood sFlt-1 levels in pregnant women who had or were suspected to have PE. In addition, PPI can also decrease the levels of endoglin and ET-1 (Saleh et al., 2017). Administration of PPI has the ability to decrease the production of certain pro-inflammatory cytokines, including IL-1b, IL-6, IL-10, and CC-motif chemokine ligand (CCL) (Onda et al., 2017). The mechanism of action of PPI to prevent preeclampsia is illustrated in Figure 7.

A cohort study conducted in South Korea found no evidence to suggest that the use of PPIsr educes the incidence of PE in pregnant women. Nevertheless, in this investigation, the dosage administered was equivalent to the therapeutic dosage prescribed for gastrointestinal issues. The precise dosage required for the prevention of PE remains uncertain (Choi and Shin, 2021). These findings are in line with the meta-analysis of Hussain et al., that the use of PPIs in pregnancy actually increases the risk of PE at any gestational age, even though the risk is very small or trivial (Hussain et al., 2022). The study conducted by van Gelder et al. concluded that administering PPI does not decrease the likelihood of developing LOP. In fact, the risk of developing this condition actually increases when PPI are used between the 17th and 33rd weeks of gestation (van Gelder et al., 2022). In another cohort study, PPIs were associated with the incidence of PE in term pregnancies. On the other hand, it was found that there was a reduced risk of PE with complications of preterm birth or with complications of birth weight that was not appropriate for the gestational age. It can be concluded that PPIs have the theoretical potential to prevent EOP (Hastie et al., 2019), currently preventative trials using PPI’s are in progress. (Poniedziałek-Czajkowska et al., 2021; Chiswick et al., 2015)

Multiple studies have confirmed the safety of using PPI during pregnancy, making them a common prescription for managing gastrointestinal issues in pregnant women. Numerous studies have shown that PPIs are safe for the fetus, and newborn, i.e., no congenital anomalies, normal birthweight distribution, and no adverse effect on Apgar scores (Chiswick et al., 2015; McDougall et al., 2022)

### Nitric-oxide donor (NO)

Nitric oxide (NO), originally identified as the endothelium-derived relaxing factor, is the main vasodilatory substance produced by the endothelium in response to chemical and mechanical stimuli. Nitric oxide is a signalling chemical that is created by a group of enzymes called nitric oxide synthases (NOS), which are dependent on calcium and calmodulin (Förstermann and Sessa, 2011). These enzymes produce nitric oxide from L-arginine. In this context, endothelial NOS is the most significant. Nitric oxide triggers the relaxation of vascular smooth muscle cells by inducing soluble guanylate cyclase (sGC). This process triggers an increase in the levels of cyclic guanosine 3′,5′-monophosphate (cGMP) inside the cells and activates protein kinases that are dependent on cGMP. PE-related endothelial dysfunction is characterized by a reduced availability of NO. Therefore, it is postulated that this will lead to an elevation in blood pressure due to the imbalance between the vasodilator and vasoconstrictor effects on the smooth muscle of the blood vessels. Nitric oxide exerts substantial inhibitory effects on platelet aggregation and activation through processes that are dependent on both cGMP and independent of it. NO also hampers the growth of vascular smooth muscle cells and the stimulation of inflammatory cells, among other tasks. Moreover, the process of S-nitrosylation, in which proteins are modified by the addition of NO, has the capability to control their activity, hence potentially impacting biological processes. Pregnant women with normotension display significant alterations in the placental S-nitroso-proteome compared to those without high blood pressure during pregnancy (Johal et al., 2014).

A systematic review of the Cochrane database, encompassing six studies, revealed a lack of conclusive evidence on the efficacy of NO donors and precursors in preventing PE or its sequelae (Meher and Duley, 2007). The review’s conclusions are mostly constrained by the insufficient sample size. The comparison of NO donor or its precursor (L-arginine) with placebo or no intervention was conducted in four studies. The available information is inadequate to establish definitive conclusions regarding the effectiveness of nitric oxide donors and precursors in preventing pre-eclampsia or its associated problems. The relative risk (RR) is 0.83 with a 95% confidence interval (CI) of 0.49–1.41. Adverse effects that occur following the administration of NO donor supplements, such as isosorbide mononitrate, may include intense headaches that are significant enough to lead to discontinuation of the treatment. Recent research indicates that isosorbide mononitrate and L-arginine have equivalent (lack of) efficacy in preventing PE (Dymara-Konopka and Laskowska, 2019). Currently, there is very limited information about the preventive effectiveness of these drugs in women who are at risk of developing pre-eclampsia.

### L-arginine

L-Arginine is a semi-essential amino acid that serves as a precursor to NO through the NOS enzymatic pathway. L-arginine is the primary precursor of NO during pregnancy, which is crucial for maintaining a sufficient blood supply to the placenta. Various studies yielded divergent results concerning alterations in L-arginine levels in PE (Wardhana et al., 2021). According to the research conducted by Tashie et al., which corresponds with other prior studies, women with PE had elevated levels of ADMA, which led to reduced levels of NO due to the inhibition of eNOS. ADMA functions as a competitive inhibitor of eNOS activity. The bioavailability of L-arginine plays a crucial role in determining the production of NO in the body. Optimal synthesis of nitric oxide (NO) occurs at physiological levels of L-arginine. The study found that the levels of L-arginine were within the normal range, however in the group with PE, the levels were comparatively elevated compared to the placebo group. This is believed to be caused by a malfunction in the transportation of L-arginine through the y + transport system or by an increase in ADMA, which hinders the uptake of L-arginine by cells through the y + transport system by acting as a competitive inhibitor (Tashie et al., 2020). These findings contrast with numerous studies that have found a decline in L-arginine levels in women with PE compared to women with normal blood pressure (Dymara-Konopka and Laskowska, 2019; Wardhana et al., 2021). The reduction in L-arginine levels is also observed in cases of severe PE (Wahyuningsih et al., 2021). The reduction of L-arginine levels, acting as a competitive inhibitor of ADMA, will lead to the impairment of NO signalling in PE (Dymara-Konopka and Laskowska, 2019).

Supplementation of L-arginine in combination with vitamins C and E prior to 24 weeks of pregnancy shown a notable decrease in the occurrence of PE as compared to the group that received a placebo (Vadillo-Ortega et al., 2011). Supplementing pregnant women with chronic HT with L-arginine can decrease the necessity for HT medications, but it does not decrease the occurrence of superimposed PE (Dymara-Konopka and Laskowska, 2019). Administering L-arginine has been shown to decrease the occurrence of PE by 74% in the study conducted by Camarena Pulido et al. (2016). These findings align with the study conducted by Nadia Taj et al., which also reported an efficacy rate of 92.3% (Taj et al., 2022). Ortega et al. conducted a RCT to compare the effects of administering food supplements including L-arginine and antioxidant vitamins with a placebo in preventing PE in high-risk groups. The occurrence of PE was notably lower in the treatment group as compared to the placebo group, with an absolute risk reduction of 0.17 (95% CI = 0.12–0.21; *p* < 0.001). Additionally, administering L-arginine in combination with antioxidant vitamins demonstrated a more effective preventive impact compared to administering antioxidant vitamins alone. The absolute risk decrease was 0.09 (95% CI 0.05–0.14, *p* = 0.004) (Vadillo-Ortega et al., 2011). A meta-analysis of 10 trials indicated that oral L-arginine supplementation was associated with a decreased risk of neonates with fetal growth restriction, preterm labor, and respiratory distress syndrome. (Goto, 2021a). Multiple studies have revealed variations in the recommended safe amount and duration of arginine supplementation for pregnant women. However, one observational study concluded that a daily dose of 30 g of arginine for a period of 90 days is considered safe during pregnancy. Nevertheless, other studies have demonstrated favorable consequences on pregnancy results through the utilization of oral arginine supplementation at low dosages (3–7 g/day) for an extended duration (Weckman et al., 2019). Table 3 displays three recent meta-analyses regarding the efficacy of L-arginine in preventing PE.

**TABLE 3:** Meta-analysis studies the effect of L-Arginine suplementation on pregnancy.

No	Authors (year)	Number of study and participants	Methods	Results
1	Chen et al. (2016)	9 trials (576 participants)	L-arginine vs. placebo	- Increase fetal birth weight - Increase gestational age on delivery - Decrease newborn RDS rates - Decrease newborn ICH - Decrease pulpability index on Umbilical Artery
2	Sagadevan et al. (2021)	7 studies (524 participants)	L-arginine vs. placebo	- Decrease PE risk (OR: 0.38; 95% CI: 0.25–0.58) - Decrease blood pressure (both systolic and diastolic) - No effect on gestational age, latency periods, and neonatal outcomes (birth weight and Apgar scores)
3	Goto (2021b)	10 studies	L-arginine vs. placebo	- Decrease pretem birth and FGR risk - Decrease newborn RDS rate - Increase fetal birth weight and gestational age on delivery - Increase newborn Apgar score - No effect on miscarriage, infection, ICH, admission to NICU, and cesarean section rates

## Future direction

Currently, only aspirin and calcium (particularly in populations with deficient calcium levels) are considered approved medications for the secondary prevention of preeclampsia. Other medications shown promise efficacy in the prevention of preeclampsia include statins and L-arginine (Akbar et al., 2024; Akbar et al., 2022). Additional medicines that have inconsistent effects include Vitamin D, metformin, proton pump inhibitors, LMWH, and NO-donors. Extensive investigations are still required to ascertain the efficacy of these medications in preventing preeclampsia. Furthermore, additional agents with the potential for preventing preeclampsia are under investigation, including immunomodulators and anti-inflammatory agents (Tacrolimus, Eculizumab, Sulfasalazine, Etanercept, Hydroxychloroquine), micronutrients (Vitamin C, Vitamin E, DHA, Folic acid, Zinc, etc.), antioxidants (Sofalcone and Resveratrol), hormones (Melatonin), Sildenafil Citrate, and herbal extracts (Nigella sativa) (Brownfoot and Rolnik, 2024; Mészáros et al., 2023b; Rahma et al., 2017; Sakowicz et al., 2023; de Alwis et al., 2020).

## Conclussion

Several pharmacological therapies demonstrate promise efficacy as preventative medicines for PE. LDA and calcium supplementation clearly represent the pivotal methods to reduce the rate of PE. Recent multicentre studies using pravastatin look very promising. Additional preventative medications, such as Metformin, LMWH, NO-donor, and L-Arginine, may be effective for particular patients with specific risk profiles (morbid obesity, placental thrombosis, etc.). Further research is required to arrive at a more individualized preventative approach for individual women with individual risk profiles and particularly also regarding timing of intervention, dose used and long-term safety.
